# Supplementing the Traditional Institutional Review Board with an Environmental Health and Community Review Board

**DOI:** 10.1289/ehp.9005

**Published:** 2006-08-14

**Authors:** Steven G. Gilbert

**Affiliations:** Institute of Neurotoxicology and Neurological Disorders, Seattle, Washington, USA

**Keywords:** autonomy, bioethics, community-based participatory research, dignity, environmental justice, institutional review board, justice, sustainability, veracity

## Abstract

**Background:**

Community-based research often involves additional ethical, legal, and social considerations beyond those of the specific individuals involved in the study. The traditional institutional review board (IRB) typically focuses on protecting the rights and ensuring the safety of the individuals involved. For projects involving community members, IRBs should be more sensitive to issues related to the broader community concerns.

**Objectives:**

The objective of this article is to discuss the concept of community-based participatory research and the shortcomings of the traditional IRBs in dealing with ethical issues associated with broader community concerns such as implications for family members, neighborhood groups, and local businesses. I examine the rationale and benefits for expanding the roles and responsibilities of review boards related to community-based issues.

**Discussion:**

I propose the development of environmental health and community review boards (EHCRBs) that combine the fundamental responsibilities and ethical concept of the traditional review boards with an expanded ethical construct of dignity, veracity, sustainability, and justice, with an added emphasis on community.

**Conclusions:**

Only by acknowledging the needs of and working with the community can we ensure ethically based and socially responsible research. An EHCRB will allow researchers and community members to more fully address their mutual interest in conducting scientific, ethical, and socially responsible research.

The purpose of this article is to consider the development of an environmental health and community review board (EHCRB). The EHCRB would function as a traditional institutional review board (IRB) but with added expertise and focus related to concerns of the community. Community-based research often involves ethical, legal, and social considerations beyond those of the specific individuals involved in the study. The current IRB system typically focuses on issues related to the individual, such as adequate informed consent, and may not be sensitive to the impact of the study on the community. For example, a study designed to identify children with elevated blood lead levels and sources of lead exposure may stigmatize the housing stock, which could affect the value of the real estate in the area. Additional conflict may arise over information directly related to specific individuals or businesses and their right to privacy. This may be in conflict with a community’s need to know and understand information essential to making good decisions. The foundation for an EHCRB is built on an ethical construct of dignity, veracity, sustainability, and justice with an added emphasis on community. The need to expand or alter the current IRB, particularly related to advances in genomics, has been recognized (National Bioethics Advisory Commission 1999; Sharp and Foster 2002). To ensure adequate consideration of community issues, I propose the establishment of EHCRBs for research or studies focused on human health in communities and workplaces.

## The Traditional IRB: History and Overview

The current or traditional IRB system of reviewing studies involving humans developed out of a recognized need to protect human study participants. The foundation of the IRB was built on acknowledging individual human rights. The Nuremberg Code and the Declaration of Helsinki (World Medical Association 2004) articulated a need for voluntary consent to any involvement in research. Furthermore, consent must be based on a full understanding of the proposed study or research. Involvement must be entirely voluntary and free of coercion. In addition, benefits from the study must outweigh any risks involved, which requires comprehensive evaluation and communication of the risks compared to the benefits. Investigators must be fully qualified and knowledgeable of all aspects of the study. And finally, participants must have the ability to withdraw at any time.

The Belmont Report (National Commission for the Protection of Human Subjects of Biomedical and Behavioral Research 1978) and others (Beauchamp and Childress 1994) went further in characterizing the rights of individuals and responsibilities of investigators or caregivers toward the client or study participant. The Belmont Report continued the focus on the individual by defining four elements: respect for autonomy, beneficence, nonmaleficence, and justice. Respect for autonomy of an individual requires that the researcher provide adequate information about the study and obtain informed consent. Beneficence and nonmaleficence refer, respectively, to maximizing the benefit and “doing good,” and to reducing risk and avoiding harm. Justice is usually characterized as being fair, but it demands consideration of broader societal issues of equity and distribution of services.

The U.S. Federal Policy for the Protection of Human Subjects (DHHS 2005, 45 CFR 46) [see Office for Human Research Protections (OHRP) 2005 for current policy information on IRBs] defines a human subject as “a living individual about whom an investigator . . . obtains data through intervention or interaction with the individual, or identifiable private information.” This definition refers only to “a living individual,” which leaves a side issue related to tissue from deceased individuals. There is additional concern for vulnerable populations such as fetuses, children, pregnant women, prisoners, and those unable to give informed consent because of a clinical condition. The focus remains on protecting the safety and ensuring the rights of the individual. Research is defined by 45 CFR 46 (DHHS 2005), as “a systematic investigation, including research development, testing and evaluation, designed to develop or contribute to generalizable knowledge.” The OHRP requirements for human testing are often referred as the Common Rule (OHRP 2006), which has been widely adopted by U.S. government agencies. This is a broad definition of research but does not include all areas of research such as that required by the U.S. Food and Drug Administration (FDA).

The FDA oversees a precautionary approach to drug and device development that requires adequate demonstration of both efficacy and safety before a new product or device can be marketed. This process generally starts with and requires extensive animal testing to evaluate efficacy and safety before proceeding to human clinical research. The FDA requires a similar IRB approval process for human research (FDA 2006, 21 CFR 50 and 56) with some minor differences with 45 CFR 46 (DHHS 2005; FDA 1998, 2000). Many of the institutional and for-profit IRBs are concerned with studies directed primarily toward development of biomedical drugs and devices (treatment/therapeutic interventions).

The current IRB system is still evolving as new biomedical treatments such as gene therapy develop. The IRB system also needs to evolve to support research designed to evaluate community-based issues, including those that focus on the assessment and prevention of adverse health effects. This type of research is often referred to as community-based research or community-based participatory research (CBPR).

## Community-Based Participatory Research

CBPR is best characterized as doing research “with” the community, not “to” the community. CBPR requires that the community be involved in all phases of the research from conception and planning to gathering data, interpreting results, and finally developing reports and action plans. A fundamental concept is that the research must benefit the community and not just the researchers. In essence, CBPR requires that adequate time be taken to involve the community and to ensure community participation in all aspects of the project.

CBPR can be summarized with the following six principles [adapted from O’Fallon and Dearry (2002)]:

- Promotes active collaboration and participation at every stage of research: All parties should share ownership of the project.
- Fosters co-learning: Researchers and participants share their knowledge and learn together.
- Ensures that projects are community driven: Projects address questions and concerns of the community, not just of the researchers.
- Disseminates results in useful terms: Project results are communicated to the participants in an appropriate and sensitive format.
- Ensures that research and intervention strategies are culturally appropriate: Investigators must be culturally sensitive to achieve the best results.
- Defines community as a unit of identity: In collaboration with participants, consideration must be given to defining the community involved in the project.

Other approaches to CBPR are also available (School of Public Health and Community Medicine 2005), as well as examples of evaluation of the effectiveness of CBPR and the challenges of environmental health research in a community (Arcury et al. 2001; Corburn 2002a; Lambert and Lane 2004; Morello-Frosch et al. 2002; Perera et al. 2002; Wing 2002). The workplace may also be considered a defined community and, as such, may require a CBPR approach when designing and interpreting studies. The workplace has unique challenges that must be addressed, such as the concerns of the business owner. The workplace varies from the traditional manufacturing area or fields in an agricultural area (Arcury et al. 2001, 2002), each of which require thoughtful consideration of unique community issues.

The traditional IRB was developed to address issues related to individuals involved in research projects. The ethical principles that support and guide the traditional IRB are also focused on the individual. To address the broader constituency of the community and workplace, a new ethical construct is required to support and guide an EHCRB.

## A New Ethical Foundation

The philosophical foundation for supporting and guiding an EHCRB moves beyond the traditional four principles established in the Belmont Report (National Commission for the Protection of Human Subjects of Biomedical and Behavioral Research 1978). Advances in scientific knowledge combined with experience are driving a reexamination of ethical issues in environmental health (Lee 2002; Sharp 2003; Wing 2003). This foundation is built on a broader ethical construct that emphasizes a fundamental commitment to greater social responsibility for individuals, communities, and businesses. Although the traditional principles of autonomy, beneficence, nonmaleficence, and justice have focused on the individual, this new construct also incorporates the community while emphasizing social responsibility and partnership.

### Dignity

Respect for autonomy is expanded to become respect for dignity and recognition of worth. This acknowledges that people, especially children, have a right to develop in an environment in which they can reach and maintain their full potential (Gilbert 2005). Children and adults have an inherent dignity and worth that we must respect and have a duty to protect. The duty is shared by parents, the community—including business and the government—and investigators. Respect for dignity incorporates the classical respect for autonomy but moves beyond that to recognize not only a right to know but a right to understand. Investigators have an obligation to expend resources to ensure that people understand the underlying issues and the implications of the work. Respect for dignity also includes respect for the dignity of the family and the community. Even more broadly, the concept of community can also be expanded to include the greater biotic community of all living things. Only by considering the combined dignity of individuals, family, and community, including the conflicting demands, can we truly address the ethical issues and promote the flourishing of all. In essence, this is respecting and working “with” people and “with” the community.

### Veracity

The complexities and judgment required when addressing issues of beneficence and nonmaleficence are supplemented by a requirement to present the facts, or veracity. Veracity is not the truth but the facts, and this means all the relevant facts. At all times, there must be a commitment to a right to know and a right to understand. It is the responsibility of the individual and even the community to decide on the truth. Providing the facts allows the individual or community to determine what is good (beneficence) and what does no harm (nonmaleficence). It is often necessary to reach a compromise between good and harm, which is a decision only informed individuals can reach.

Veracity directly addresses the right to know and the right to understand. Those with the knowledge and the facts have the responsibility and obligation to provide the facts along with appropriate information to ensure understanding. Sufficient resources must be allocated to address the community’s and individual’s need for factual information. Veracity also relates to a community’s need to know certain information that individuals or businesses would rather not share. Only with this information can the dignity of a community be respected and appropriate decisions made. The principle and responsibility of veracity are shared by investigators and community members. All parties must work together to share information and develop a mutual understanding of the project goals and outcomes.

### Sustainability

An additional element of this ethical framework is sustainability. Advances in science and technology combined with the expanded human population require that our actions be sustainable over the long term. Although one person may be able to pick a flower from a meadow with little appreciable change, a million cannot each pick a flower without harming the beauty and function of the meadow. Similarly, we must examine our actions and approaches to ensure that they are sustainable over the long term. Poisoning children with lead or fish with mercury harms the individual, degrades the biotic community, and is not sustainable. Ultimately, harming our children degrades the future generations of our communities. Our efforts must sustain the individual as well as the broader ecologic and local community, for they are inextricably linked. Further, we must address the sustainability of the business community along with that of the local neighborhoods. This requires that the needs and requirements of the business community be considered in collaboration with neighborhood committees.

Another aspect of sustainability in CBPR is building and nurturing capacity within a community. This starts by sharing information and involving people in a study—in other words, doing the project “with” the people and the community. Building capacity within a community helps to address the sustainability of that community by empowering people with knowledge. Knowledge enables people to define their own truths and develop sustainable communities. Thoughtful CBPR projects consider the complexity of a community and incorporate sustainability into study design and execution.

### Justice

Justice is not only about ensuring fairness but also about the compromises that are inevitably required when addressing dignity, veracity, and sustainability. The concept of justice must be expanded to include what is just for the community. An individual or business may want to keep certain information private or confidential, but this may not be just for the community. We must acknowledge and address the conflicts between what is just for an individual and what is just for the community. The community may not be sustainable without considering information and needs related to an individual or business. There are also issues related to environmental justice. The U.S. Environmental Protection Agency defines environmental justice as “the fair treatment and meaningful involvement of all people regardless of race, color, national origin, or income with respect to the development, implementation, and enforcement of environmental laws, regulations, and policies” (U.S. Environmental Protection Agency 2005). The concept of environmental justice clearly supports the establishment of an EHCRB and evaluation of community needs (Lee 2002).

**Figure 1:**
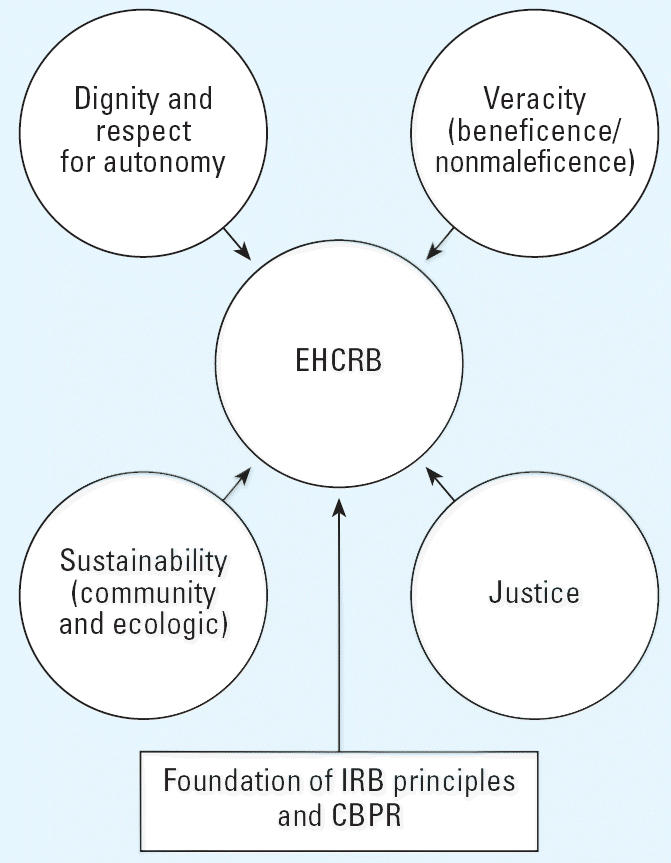
Key elements of EHCRBs.

CBPR studies or projects that collect data from identifiable human subjects require IRB approval. The principles that form the foundations of community-based research are sufficiently different from standard biomedical research protocol that a specialized IRB would be advantageous for all groups involved. The EHCRB concept is summarized in Figure 1.

## Environmental Health and Community Review Board

An EHCRB would assume the traditional duties and responsibilities of an IRB but would also have an ethical responsibility to the community in which the project was taking place. A CBPR project has many unique characteristics that are not encountered in a randomly controlled study of a new drug. Evaluation of a CBPR project requires training, knowledge, and sensitivity to the additional factors associated with these projects. In essence, an EHCRB must go beyond consideration of only the legal requirements and be cognizant of the ethical and social issues involved in community-based research. The EHCRB may serve as a bridge or forum that would help the investigators, individuals, and communities involved achieve a mutually beneficial project. Native American nations have a cultural tradition that supports a community-based consultation approach that is similar to an EHCRB concept and has been used to deal with environmental issues (Arquette et al. 2002). The EHCRB would also be supportive of communities actively working to integrate health-related information into social issues (Brown et al. 2004).

An EHCRB would go beyond considering the rights of individuals directly involved in a study to include the implications for the family and community. For example, should there be an informed consent process for the family and community? This also requires a process of defining the community. The community might be a physical area defined by chemical contamination. The evaluation of individuals for exposure to these chemicals may have health or economic implications for everyone living within the area. If some of the properties are rented, there may be social and legal implications for the property owners. The business community may have different concerns. The EHCRB must ensure that the investigators have adequately defined and identified the community and stakeholders along with their potential concerns.

Once the community is identified, an EHCRB would determine whether investigators have adequately consulted the community and incorporated their concerns into the project design. This would include ensuring not only a right to know but also a right to understand the issues involved. The investigators need to demonstrate a plan for community involvement in the project at the very earliest stages. The research protocol should demonstrate how the community will participate in the study and will continue to be involved and informed throughout the study. Consultation with a community helps to direct the study and define the needs of the community (Corburn 2002b). Sufficient resources, including time, must be devoted to communication with the community. An important goal of a CBPR project is to help build capabilities and capacity in the community to address issues related to the investigation. An experienced EHCRB would ensure that the project leaves behind more that it takes away from the community.

Community-based research often has important direct and indirect implications for the individuals and community. Individuals, families, and communities may have different interests and levels of concerns that must be addressed throughout the phases of a study. There may be different privacy needs or right to know for an individual, family, community, or business. For example, examining of blood lead levels in one child may have implications not only for other family members but also for other individuals and families living in that building. Similarly, a child’s right to know needs to be addressed along with a parent’s right to information. This could be an issue in testing for drug use. In studies involving environmental chemicals, there is usually no informed consent regarding the exposure to the chemicals, which may have legal and business considerations. Although respect for privacy is important, in some circumstances too much privacy may also hinder the goals of the community to address issues. For example, it may be very important for all the children living within a building to know that there is danger of lead exposure. A goal of the EHCRB is to work with the investigators and community to ensure that all implications of the study are adequately addressed and find a balance that protects the individuals as well as the community.

Projects or investigations based on a defined workplace often have additional considerations that must be addressed. The business owner may be concerned about the legal and regulatory implications of the study results. Even if the study is conducted outside the workplace, there may be implications for the business owner. For example, what are the consequences of discovering that a worker is inadvertently contaminating the home with chemicals originating from the workplace? The business owner or manager may want to protect revenue or profit and not wish to make additional investments in areas related to worker health and safety. These are important considerations in a capitalist economic system where business owners and managers are rewarded by externalizing costs and increasing profits. A worker may also not want to raise a workplace issue for fear of losing a job or creating a difficult situation at the workplace. An EHCRB must be sensitive to the issues and help the investigators in addressing issues related to the workplace.

Many members of an EHCRB would be similar to those in a traditional IRB but will need added sensitivity and experience with community-based research. It is important to have several community members on the EHCRB. To ensure that communities feel that the EHCRB is working with them, the board should be established within a community organization, not at a local university or college. This may be a first step in community capacity building. Meetings of the board should be held at times convenient for community members, to encourage involvement. There of course must be adequate training of all members in the goals and approach of community-based research as well as the requirements and responsibilities of IRB members, including protection of human subjects.

## Summary of EHCRB Responsibilities and Concerns

A summary of some of the EHCRB responsibilities and concerns is provided below. There is of course some overlap because it is difficult to seamlessly divide the responsibilities of an EHCRB into unique categories.

### Community and stakeholders

- Define the community and stakeholders
- Ensure adequate representation of the community
- Consider implications for family and community: Is there a need for informed consent for family or community? And is there potential harm at the health or economic level?
- Consider the rights of property owners and tenants
- Include economic issues—consideration of concerns of the business community
- Consider issues related to workers and business owners.

### Consultation with the community

- Consult community members, work with advisory boards
- Commit resources to the right to know and the right to understand
- To facilitate researchers’ efforts, become educated on community and family risks and needs
- Build capacity within the community
- Share data and results with the community before release to media or scientific publication
- Address issues of prevention as well as treatment.

### Implications for individuals and the community

- Recognize differences in and levels of concerns for individuals/families/communities
- Recognize that studies may involve situations were there was no informed consent for exposures
- Consider children’s right to know and to privacy versus parents’ need to know
- Recognize privacy needs of individuals, families, communities, business units
- Recognize that too much privacy (lack of transparency or sequestering information) hinders community decision making
- Support and protect community interests
- Consider economic consequences to individual, family, and community.

### Workplace-based projects

- Recognize owners’ motivation to externalize costs
- Consider motivation to increase revenue and profits
- Recognize legal and regulatory issues.

### EHCRB membership and conduct

- Include community members, scientists, and ethicists
- Establish the board within a community, not within an institution
- Hold meetings convenient for the community
- Include roles and responsibilities of a current IRB
- Expand role to include elements of dignity, veracity, sustainability, and justice.

## Conclusion

The concept of an EHCRB is based on the need to recognize an ethical and social responsibility not only to the individual but also to the community. The foundation for the concept is built on the principles of dignity, veracity, sustainability, and justice. These principles are defined with added dimension and inclusion of the community. This concept also recognizes that there are situations in which the rights of the individual and those of the community conflict. The EHCRB assumes the traditional role of an IRB in protecting the human subjects but also addresses the rights and concerns of the community. Only by defining and addressing the needs of the community can we ensure ethically based and socially responsible research.
